# Mutational landscape of homologous recombination‐related genes in small‐cell lung cancer

**DOI:** 10.1002/cam4.5148

**Published:** 2022-08-26

**Authors:** Shuo Wu, Yao Zhang, Yan Zhang, Liz‐han Chen, Hai‐feng Ouyang, Xi Xu, Ying Du, Xin‐yu Ti

**Affiliations:** ^1^ Department of Pulmonary and Critical Care Medicine, Xijing Hospital Fourth Military Medical University Xi'an Shanxi China; ^2^ Department of Pulmonary Medicine, Xi'an International Medical Center Hospital Xi'an Shanxi China; ^3^ Genecast Biotechnology Co., Ltd Wuxi Jiangsu China

**Keywords:** biomarker, homologous recombination deficiency, immunotherapy, small‐cell lung cancer

## Abstract

**Background:**

Homologous recombination deficiency (HRD) is a well‐known biomarker which could predict poly‐ADP ribose polymerase 1 (PARP) inhibitor and platinum drug response. As an aggressive cancer, small‐cell lung cancer (SCLC) is sensitive to platinum drugs, but relapse occurs rapidly. Herein, we aim to illustrate the genomic alteration patterns of homologous recombination repair (HRR)‐related genes in a Chinese SCLC cohort and further analyze the relationship among HRR gene mutations and known biomarkers of immune checkpoint inhibitor (ICI) response, including tumor mutation burden (TMB) and programmed cell death‐ligand 1 (PD‐L1) expression.

**Methods:**

Next‐generation sequencing (NGS)‐based target capture sequencing of 543 cancer‐related genes was performed to analyze the genomic profiles of 133 Chinese SCLC patients, and TMB was calculated. PD‐L1 expression was evaluated in 90 out of 133 patients using the SP142 PD‐L1 immunohistochemistry assay.

**Results:**

Among the 133 patients with SCLC, 47 (35.3%) had HRR gene mutations. *ATM* (8.3%) was the most frequently mutated HRR gene in the cohort, followed by *NBN* (4.5%)*.* Pathogenic somatic and germline mutations of HRR genes were identified in 11 (23.4%) and 4 (8.5%) patients, respectively. HRR gene mutations cooccurred with *KMT2D* gene mutations. There were several differences in genomic alterations between patients with HRR gene mutations (HRR‐Mut) and without HRR mutations (HRR‐WT). The results revealed that *TP53* and *RB1* were commonly mutated genes in both groups. Mutations in the *KMT2D* gene and genes in the RTK‐RAS pathway occurred more frequently in the HRR‐Mut group. Furthermore, we found that mutations in HRR genes were associated with high TMB (Wilcoxon, *p* = 0.048), but there was no correlation of HRR gene mutation status with PD‐L1 expression.

**Conclusions:**

We exhaustively describe the genomic alteration profile of Chinese SCLC patients and provide further evidence that HRR gene mutations are prevalent in SCLC patients.

## Graphical abstract

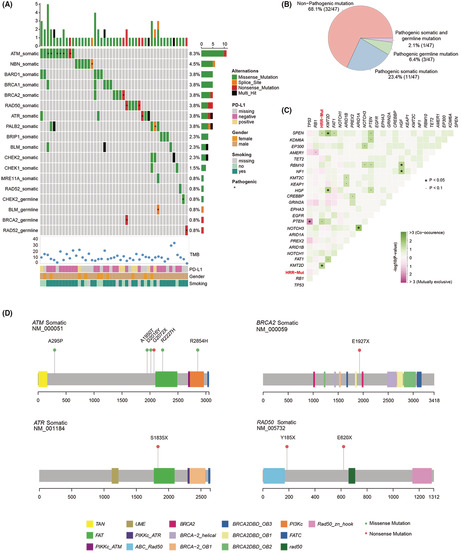

## 1 INTRODUCTION

Approximately 15% of all lung cancers are Small cell lung cancer (SCLC) and SCLC is a very aggressive disease with widespread metastases at a early stage.^1^ A large proportion of newly diagnosed SCLC patients were extensive‐stage disease, which is considered incurable.^2^ Systemic chemotherapy with platinum‐based regimens is the standard of care for SCLC, which has not changed for the past 30 years. Standard treatment with or without immunotherapy was also used for SCLC patients with metastatic disease. Although the response rates to these therapies for SCLC are above 60%, even in patients with metastatic disease, which is very acceptable, the duration of response is not very long.^3^ New therapies urgently need to be found to improve the survival of SCLC patients.

Homologous recombination deficiency (HRD) is a kind of DNA repair defect, while several cellular processes relay on homologous recombination, including the DNA double‐strand breaks (DSBs) repair and stalled DNA replication forks recovery.^4^ Tumors with HRD are sensitive to platinum chemotherapy, which generates interstrand cross‐link, and inhibitors of the poly‐ADP ribose polymerase 1 (PARP1),which is an enzyme for DNA repairing.^5^ Breast cancer susceptibility protein 1 and 2 (*BRCA1* and *BRCA2*) genes are the best known HRD‐related genes, and other genes, such as Ataxia‐telangiectasia mutated (*ATM*), BRCA1‐Associated RING Domain 1 (*BRAD1*), and BRCA1 interacting protein C‐terminal helicase 1 (*BRIP1*), have been found to be involved in homologous recombination and related pathways.^6^ Therapies targeting HRD, such as PARP inhibitors, have been widely used in breast cancer, ovarian cancer, and prostate cancer.^7^, ^8^, ^9^, ^10^ BRCA mutation and HRD status have been exploited as biomarkers of PARP inhibitor response. The genomic instability and sensitivity to cytotoxic chemotherapy of SCLC make PARP inhibitors a promising option in research on targeted therapy. In addition, immune checkpoint inhibitors (ICIs) combined with platinum drugs have recently been shown to have the potential to improve survival outcomes in SCLC patients, and it has been reported that HRD is a predictor of response to immunotherapies.^11^, ^12^

In our present study, we first described the mutation landscape and characteristics of HRR genes in Chinese SCLC patients and further analyzed the relationships among HRR gene mutations, tumor mutation burden (TMB) and programmed cell death‐ligand 1 (PD‐L1) expression. Collectively, our investigation indicated that HRR gene mutations were enriched in the Chinese population. We also discussed the potential of HRD status as a predictor of response to ICIs in SCLC.

## 2 MATERIALS AND METHODS

### 2.1 Patient information and HRR gene definition

Pathologically confirmed Chinese SCLC patients (*n* = 133) were enrolled in this study from the First Affiliated Hospital of the Air Force Medical University. To identify both somatic and germline mutations, formalin‐fixed paraffin‐embedded (FFPE) tumor tissues were collected for next‐generation sequencing (NGS)‐based target capture sequencing with a gene panel that included 543 cancer‐related genes, as well as the matched blood samples. In our study, patients with at least one mutation in any of 15 HRR genes were divided into HRR‐Mut group and the 15 HRR genes included *ATM*, ataxia‐telangiectasia and Rad3‐related kinase (*ATR*), *BARD1*, BLM RecQ like helicase (*BLM*), *BRCA1*, *BRCA2*, *BRIP1*, Checkpoint Kinase 1 (*CHEK1*), Checkpoint Kinase 1 (*CHEK2*), MRE11 Homolog, Double Strand Break Repair Nuclease (*MRE11A*), nibrin (*NBN*), Partner And Localizer Of BRCA2 (*PALB2*), RAD50 Double Strand Break Repair Protein (*RAD50*), RAD51 Recombinase (*RAD51*), and DNA Repair Protein RAD52 Homolog (*RAD52*).

### 2.2 DNA extraction and sequencing

To identify somatic or germline single‐nucleotide variants (SNVs) and insertion and/or deletion (indel) mutations, FFPE tumor samples were used to detect mutations and matched blood samples were used as controls. A blackPREP FFPE DNA Kit (Analytik Jena, Germany) was used to perform DNA isolation from the FFPE segments. DNA of controls were extracted from peripheral blood lymphocytes by using Tiangen Whole Blood DNA Kit (Tiangen), and the lymphocytes come from Whole blood by centrifuging (1600*g*) for 10 min at room temperature. A Covaris M220 focused ultrasonicator (Covaris) was used for genomic DNA fragmentation (150–200‐bp segments) and a KAPA HTP library preparation kit for the Illumina platform (KAPA Biosystems) was used to construct the DNA library. Then the DNA library (NimbleGen SeqCap EZ Library; Roche) was captured with a 543‐gene panel involving the sequences of major tumor‐associated genes, and sequenced by an Illumina HiSeq X‐Ten instrument. These processes were all performed according to the manufacturers' instructions.

### 2.3 Variant calling

VarScan2 (v2.4.2) was used to call somatic cell SNVs, and the variants were needed to satisfy the following filtering criterion: (i) sequencing coverage: control >50× and tumor >100×; (ii) mutated allele frequency >2%; (iii) number of mutant allele reads >2; (iv) SNVs and indels located in exonic regions; and (v) allele frequency <0.5% in the Exome Aggregation Consortium (ExAC) database or Genome Aggregation Database (gnomAD) database. The exclusion criteria for germline variants in the tumor sample or the blood sample was allele frequency of variant <0.2. The determination of pathogenicity of germline gene variants referred to the ClinVar database (https://www.ncbi.nlm.nih.gov/clinvar/), and only pathogenic or likely pathogenic germline variants were considered deleterious germline HRR gene mutations.

### 2.4 Analysis of TMB

TMB (mutations/Mb) was calculated using a previously reported algorithm.^13^ TMB was evaluated in patients with matched tumor and control samples and was measured in mutations per Mb. The median was defined as the cutoff point to stratify patients into two groups (high TMB group and low TMB group) according to a reported study.^14^

### 2.5 Analysis of PD‐L1 expression

The expression of PD‐L1 was assessed through immunohistochemistry staining and determined as a proportion^15^; the analyses were performed in 90 out of 133 SCLC patients. PD‐L1 expression was analyzed by the SP142 Kit (ZSGB‐B IO, Beijing, China), according to manufacturers' instruction. PD‐L1 positivity was defined as more than 1% of tumor immune cells staining positive, while those samples with less than 1% of tumor cells or immune cells staining positive or with no staining were considered negative.

### 2.6 Statistical analysis

All statistical analyses were performed with R version 4.1.1 software (https://www.r‐project.org/, Institute for Statistics and Mathematics). Comparisons between 2 categorical variables were analyzed by Fisher's exact test, and comparisons between 2 continuous variables were analyzed by and the Mann–Whitney U test. *p* < 0.05 were considered statistically significant.

## 3 RESULTS

### 3.1 Mutational landscape of HRR genes in Chinese SCLC patients

**FIGURE 1:**
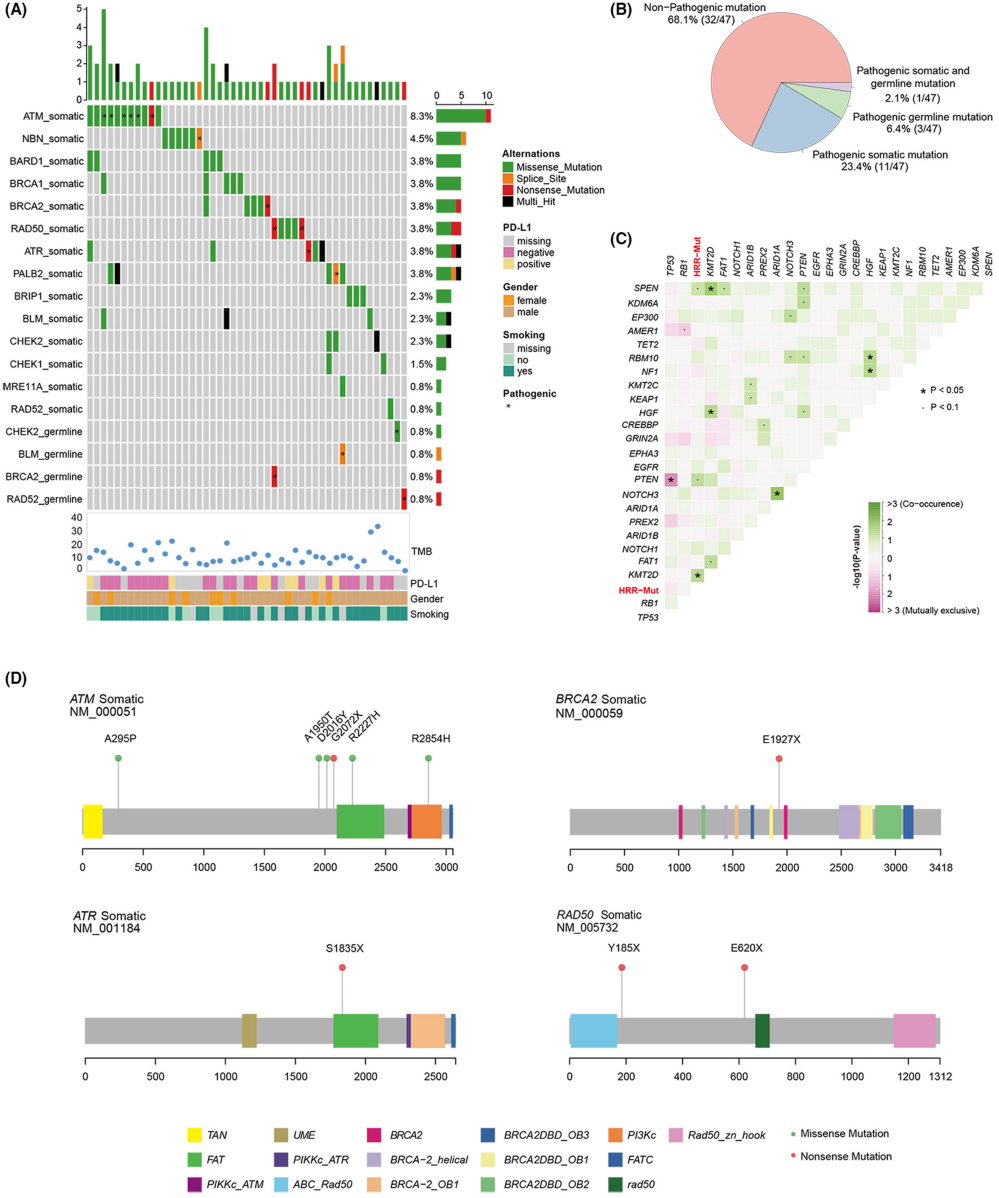
Mutational profile of HRR genes in Chinese patients with SCLC. (A) Landscape of HRR genes in Chinese SCLC patients (* indicates pathogenic HRR gene mutation). (B) Overall distribution of HRR mutation types in this cohort. (C) Cooccurring and mutually exclusive somatic mutations in this cohort. *p* values were calculated with Fisher's exact test (*p < 0.05, •*p* < 0.1). All 63 somatic mutations in HRR genes are marked as HR‐Mut. (D) Lollipop graph of the pathogenic somatic HRR mutations detected in this cohort.

Of the 133 SCLC patients, 35.3% (47/133) of patients exhibited genomic alterations in HRR genes (Figure 1A). We identified 63 HRR gene mutations in 47 patients, and most of them (88.3%) were missense mutations (Figure S1A,B). *ATM* (8.3%) was the most frequently mutated HRR gene in the Chinese SCLC patients, followed by *NBN* (4.5%), *BARD1* (3.8%), *BRCA1* (3.8%), *BRCA2* (3.8%), and *RAD50* (3.8%). In these HRR‐Mut patients, pathogenic mutations of HRR genes were identified in 15 patients, including 11 patients (23.4%) with somatic mutations, 3 patients (6.4%) with germline mutations, and 1 patient (2.1%) carrying both germline and somatic pathogenic variants (Figure 1B). HRR gene mutations significantly co‐occurred with *KMT2D* gene mutations in SCLC patients (Figure 1C). Out of all 63 HRR gene mutations, 16 mutations were pathogenic. Twelve of the mutations were somatic and occurred in 6 genes, including *ATM*, *NBN*, *BRCA2*, *RAD50*, *ATR*, and *PALB2* (Figure 1A,D). The specific locations and amino acid changes of these mutations except for 2 splicing site mutations were determined (Figure 1D). The other 4 pathogenic mutations were germline mutations of the *CHEK2*, *BLM*, *BRCA2*, and *RAD52* genes, and their detailed information is shown in Figure S1C and Table S1. No significant differences in clinical characteristics, including sex, smoking history, tumor stage, and family history of cancer, were found between HRR‐Mut and HRR‐WT patients (Table 1).

**TABLE 1:** Clinical characteristics of HRR‐Mut and HRR‐WT patients

	HRR Mut (*n* = 47)	HRR WT (*n* = 86)	*p* value
Age (years, median [IQR])	64.0 [58.5, 72.0]	63.0 [57.0, 70.8]	0.444
Gender (%)			
Female	10 (21.28)	21 (24.42)	0.8453
Male	37 (78.72)	65 (75.58)	
Smoking (%)			
Yes	32 (68.09)	53 (61.63)	0.7442
No	10 (21.28)	23 (26.74)	
Unknown	5 (10.64)	10 (11.63)	
Stage (%)			
I	0 (0.00)	2 (2.33)	0.9273
II	0 (0.00)	2 (2.33)	
III	5 (10.64)	9 (10.47)	
IV	7 (14.89)	12 (13.95)	
Unknown	35 (74.47)	61 (70.93)	
Family history (%)			
Yes	13 (27.66)	26 (30.23)	0.7698
No	24 (51.06)	46 (53.49)	
Unknown	10 (21.28)	14 (16.28)	

### 3.2 Genetic alterations between the HRR‐Mut and HRR‐WT patients

**FIGURE 2:**
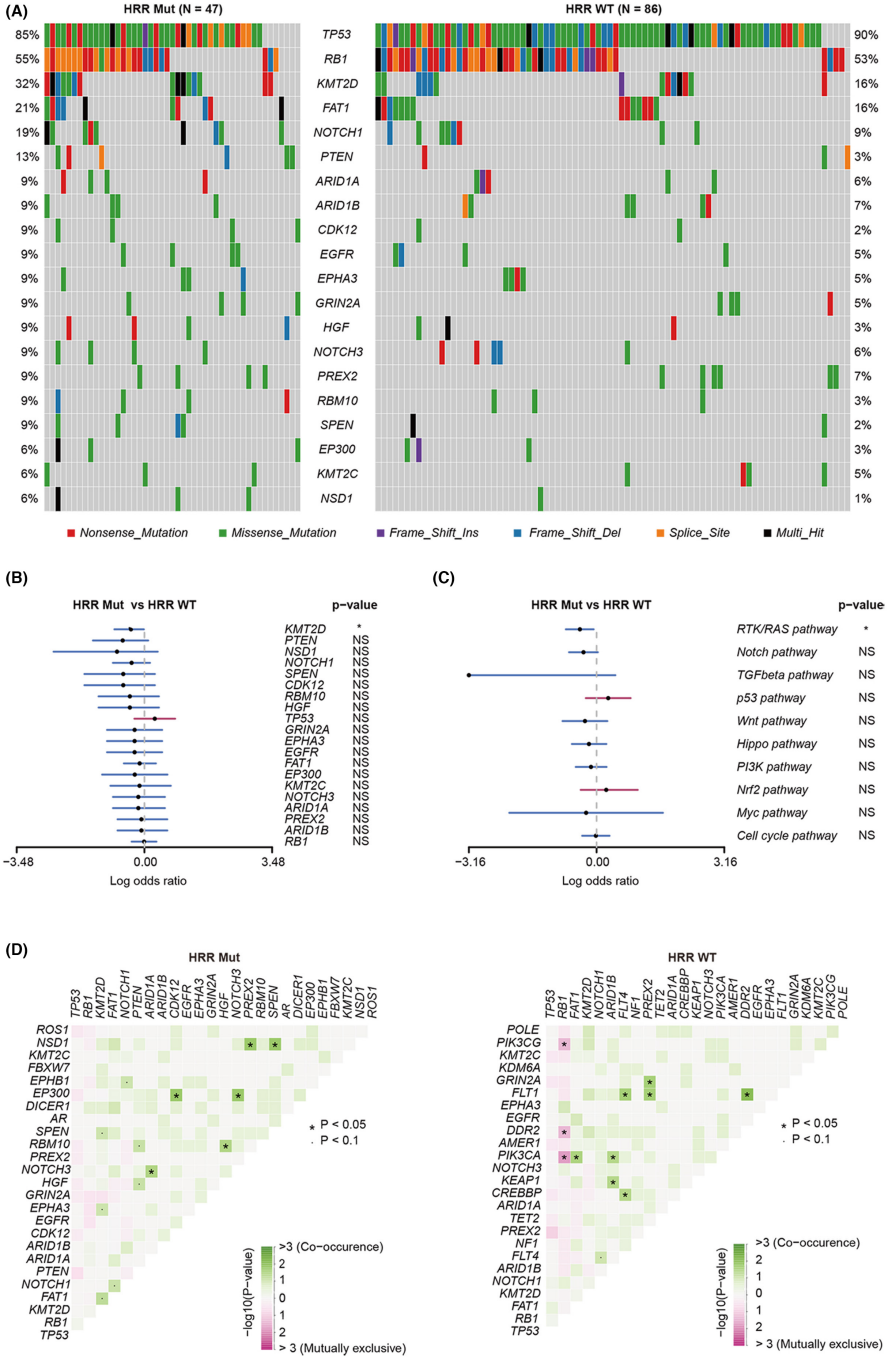
Comparison of genomic alterations between the HRR‐Mut and HRR‐WT groups of Chinese SCLC patients. (A) Mutational landscapes of the HRR‐Mut and HRR‐WT groups. (B) Forest plot showing enrichment of gene mutations in the HRR‐Mut and HRR‐WT groups by logarithmic odds ratio (*p < 0.05). The x‐axis shows the log odds ratio. (C) Forest plot showing enrichment of pathway mutations in the HRR‐Mut and HRR‐WT groups by logarithmic odds ratio (*p < 0.05). The x‐axis shows the log odds ratio. (D) Cooccurring and mutually exclusive somatic mutations in the HRR‐Mut and HRR‐WT groups. *p* values were calculated with Fisher's exact test (*p < 0.05, •*p* < 0.1).

To better understand the genomic alteration profile of SCLC patients with the HRD phenotype. we compared the gene mutation frequencies between the HRR‐Mut and HRR‐WT groups. *TP53* and *RB1* were the most commonly mutated genes in both the HRR‐Mut and HRR‐WT groups. These two genes were also identified as the top significantly mutated genes in Western SCLC patients.^16^ The *KMT2D* gene was more frequently mutated in HRR‐Mut than in HRR‐WT patients (Figure 2B), and its mutation frequency was higher than that reported in Western SCLC patents.^17^ Similarly, *RTK/RAS* pathway mutations were also more frequent in HRR‐Mut patients (Figure 2C). The mutually exclusive and cooccurring gene mutations were different between HRR‐Mut and HRR‐WT patients (Figure 2D). For instance, *RB1* gene mutations were mutually exclusive with PI3KCA gene mutations in HRR‐WT patients but not in HRR‐Mut patients. The genomic alteration profiles of SCLC patients with and without pathogenetic HRR gene mutations are shown in Figure S2A. There were no significant differences between the pathogenic mutation and nonpathogenic mutation groups in gene and pathway mutation frequencies (Figure S2B,C).

### 3.3 Associations between HRR gene status and TMB or PD‐L1 expression

**FIGURE 3:**
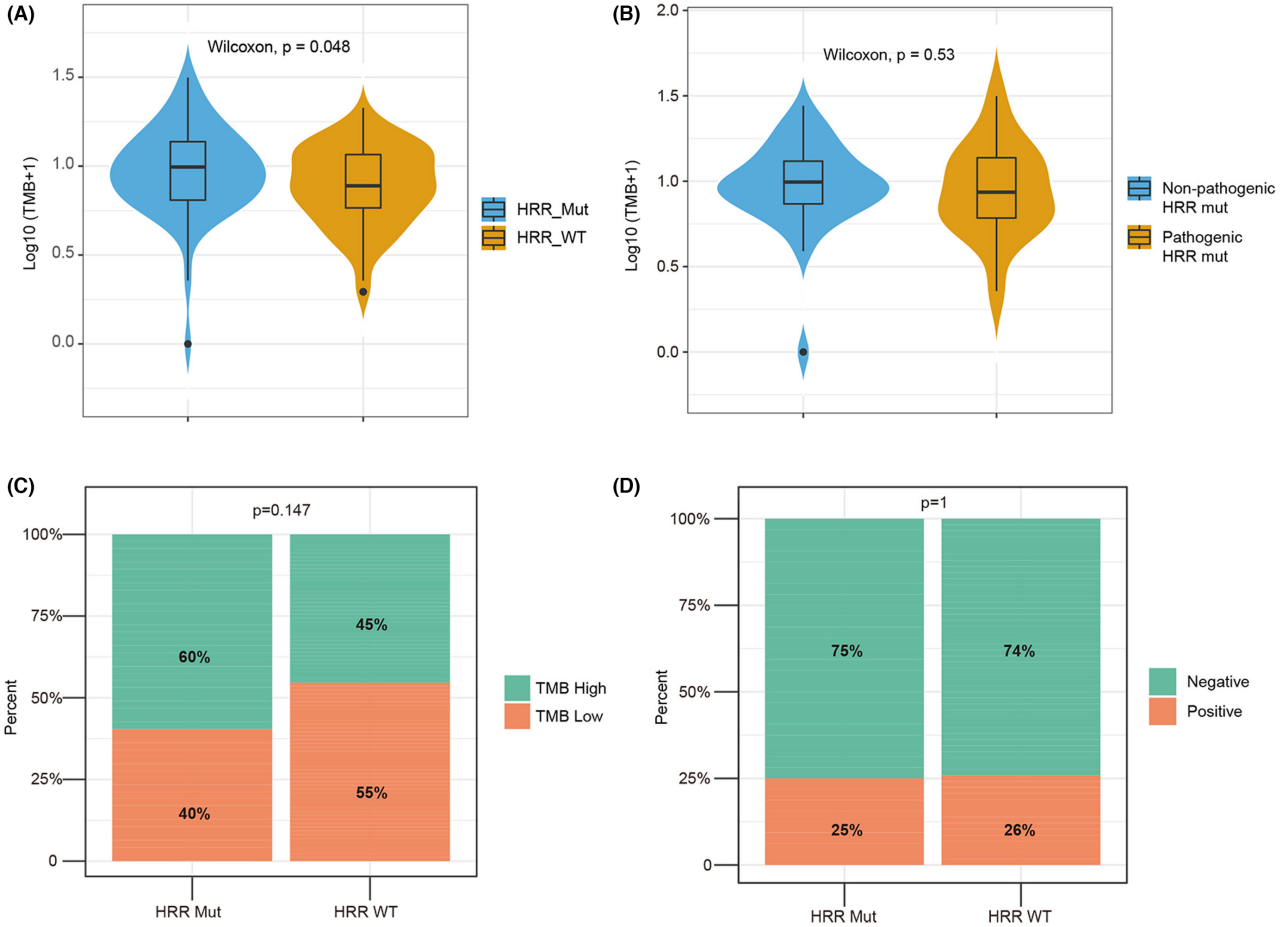
HRR gene mutations were associated with a high TMB but not with PD‐L1 expression in this cohort. (A) Comparison of TMB levels between the HRR‐Mut group and HRR‐WT group. (B) Comparison of TMB levels between patients with and without pathogenic HRR gene mutations in HRR‐Mut group. (C) Bar plots of the percentages of patients with high TMB in the HRR‐Mut group and in the HRR‐WT group (with TMB status determined using the median TMB as the cutoff point). (D) Bar plots of the percentages of PD‐L1‐positive patients in the HRR‐Mut group and in the HRR‐WT group.

SCLC exhibits an extremely high TMB.^16^, ^18^, ^19^ The median TMB was 7.62/Mb in our cohort, and the TMB of 31.6% (42/133) of patients was higher than 10/Mb. We then compared the TMB between the HRR‐Mut and HRR‐WT groups. The results showed that the TMB of the HRR‐Mut group was significantly higher than that of the HRR‐WT group (Wilcoxon, *p* = 0.048) (Figure 3A). However, there was no difference in TMB between patients with and without pathogenic HRR gene mutations in the HRR‐Mut group (Figure 3B). In previous studies, it was reported that the median TMB could predict the prognosis of SCLC patients treated with ICI therapy.^14^, ^20^ Therefore, we used the median as the cutoff point to divide the 133 SCLC patients into two groups (high TMB group and low TMB group). We found that in the HRR‐Mut group, the proportion of patients in the high TMB group was higher than that in the HRR‐WT group (Figure 3C). In the four patients with germline HRR gene mutations, the TMB of three of them was higher than the median TMB of this cohort (8.89/Mb, 12.71/Mb and 30.51/Mb), and the TMB of the other patient was slightly lower (3.85/Mb).

In our study, PD‐L1 expression status was detected in 90 patients; of these, 46.7% (42/90) were positive, but there was no correlation of HRD phenotype with PD‐L1 expression in either the HRR‐Mut or the HRR‐WT group (Figure 3D).

### 3.4 Comprehensive profile of SCLC patients with different TMBs, PD‐L1 statuses, and HRD statuses

**FIGURE 4:**
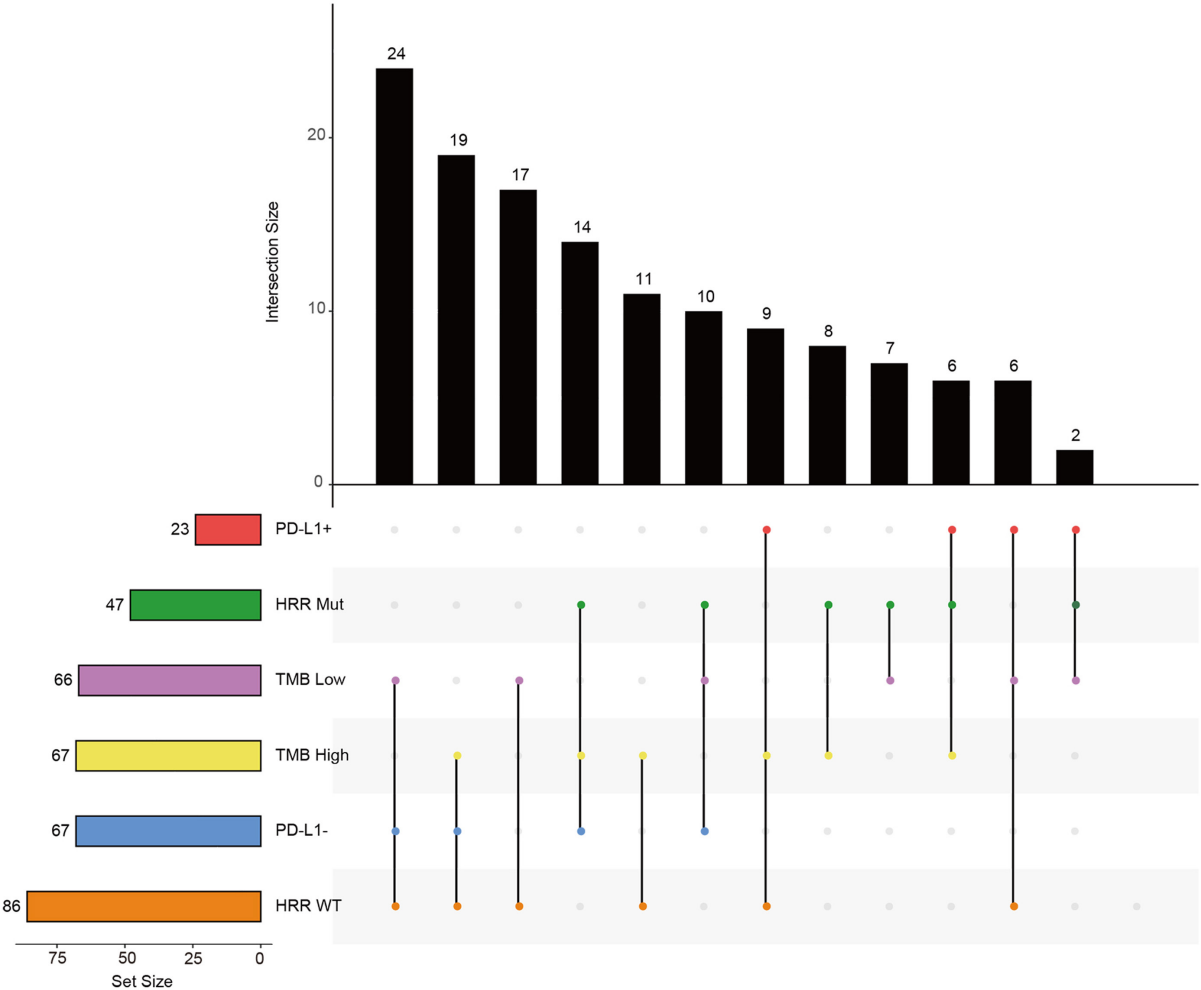
Venn diagram showing the comprehensive profile of SCLC patients with different TMBs, PD‐L1 statuses, and HRD statuses. PD‐L1+, PD‐L1 positive; PD‐L1‐, PD‐L1 negative; HRR‐Mut, patient with mutated HRR genes, HRR‐WT, patient with wild‐type HRR genes; high TMB, TMB equal to or higher than the median TMB of the cohort; low TMB, TMB lower than the median TMB of the cohort.

In previous studies, TMB, PD‐L1 expression, and HRD phenotype could predict the prognosis of advanced cancer patients who underwent ICI treatment.^11^, ^12^, ^15^ Therefore, we used a Venn diagram to comprehensively visualize SCLC patients in terms of these three factors (Figure 4). Five percent (6/133) of patients had high TMB, PD‐L1(+) and HRR gene mutations, 7% (9/133) of patients had only high TMB and PD‐L1 positivity, and 2% (2/133) of patients had only HRR mutations and PD‐L1 positivity. These three factors could precisely stratify SCLC patients to identify which might benefit from ICI or PARP inhibitor therapy or a combination of these two therapies, but this potential utility needs to be verified in future studies (Figure 4).

## 4 DISCUSSION

Here, our study analyzed the mutational landscape of HRR genes in Chinese SCLC patients for the first time and explored the differences in genomic alterations between SCLC patients with and without HRR gene mutations. The aim of our study was to clarify the HRR gene profile in Chinese SCLC patients and discuss the potential of HRR gene mutation status as a biomarker for response to PARP inhibitors, ICI therapy, or a combination of these two types of treatments.

Our results indicated that 35.3% (47/133) of patients with SCLC carried HRR mutations, 11.3% (15/133) of whom carried pathogenic HRR mutations. Furthermore, 4 germline pathogenic HRR gene mutations were detected in this cohort. Only the *BRCA2* gene was also found to carry germline pathogenic mutations in Western SCLC patients.^21^ Germline mutations of *CHEK2* and *BLM* have been reported to be associated with several types of cancer, including prostate cancer,^22^, ^23^ uterine serous carcinoma,^24^ and breast and ovarian cancer.^25^ Several cellular processes were concerned about HRR pathway, include but not limited to DNA DSBs reparation and stalled DNA replication forks recovery.^4^, ^26^ DNA‐damaging therapies such as cisplatin or PARP inhibitors might have good response in Tumors with HRR deficiency. Earlier findings showed that the response rate of SCLC patients to chemoradiotherapy was very good.^27^ Additionally, several recent studies have explored the efficacy of PARP inhibitors in SCLC patients. It was reported that veliparib plus platinum chemotherapy followed by veliparib maintenance therapy as a first‐line treatment displayed improved progression‐free survival (PFS) for extensive‐stage small‐cell lung cancer (ES‐SCLC) with an acceptable safety profile.^28^ Another phase 3 study showed that niraparib modestly improved PFS in patients with platinum‐responsive ES‐SCLC.^29^ According to these studies, HRR gene mutation status has the potential to be a biomarker for identifying SCLC patients who might respond to PARP inhibitors.

By comparing the genomic alterations of the HRR‐Mut and HRR‐WT groups, we found that the mutation frequency of the *KMT2D* gene was much higher in the HRR‐Mut group. The *KMT2D* gene, also known as the *MLL2* gene, plays a key role in regulating transcriptional enhancer function with tmethyltransferase activity toward histone H3 lysine 4 (H3K4).^30^ Recently, it was reported that a high‐frequency of *KMT2C/D* mutations could be a biomarker for PARP inhibitor therapy response in non‐small‐cell lung cancer (NSCLC) and other cancer types.^31^ This result suggested that both *KMT2D* and HRR gene mutations might indicate a better response to PARP inhibitors in SCLC patients.

SCLC has a high mutation load and high immunogenicity, thus indicating a likely higher response to immunotherapy. Several studies have shown the promising efficacy of immunotherapy in SCLC patients. The IMpower133 study showed that atezolizumab plus chemotherapy resulted in significantly longer overall survival and PFS than chemotherapy alone as the first‐line treatment for ES‐SCLC.^32^ However, there is still a lack of effective biomarkers in SCLC. Currently, PD‐L1 expression and TMB are thought to be important biomarkers of response for ICI therapy. In the present study, we observed a high TMB (median TMB = 7.62/Mb) in the SCLC cohort, and HRR‐Mut patients showed higher TMB than HRR‐WT patients. This finding is consistent with previous studies, which indicated that pathways related to HRR are associated with high TMB, such as DNA damage response (DDR), mismatch repair (MMR), or base excision repair (BER).^33^

In addition, we also observed that 25.6% of patients had positive PD‐L1 expression in 90 SCLC patients, and the proportion of positive PD‐L1 expression was similar to that reported in an SCLC meta‐analysis (22%).^34^ In a previous study, of 9321 colorectal cancer tumors, 1270 (13.6%) were HRD, and in the microsatellite‐stable/proficient mismatch repair (MSS/pMMR) subgroup, HRD tumors had a larger proportion of PD‐L1 positive tumors than non‐HRD tumors.^35^ A correlation of HRD status and PD‐L1 expression was not observed in our study. This may be because of the different cancer types. However, the absence of a correlation of HRD status with PD‐L1 expression in these SCLC patients makes it possible to use these two factors together as indicators to predict the response to ICI therapy, similar to HRD status and TMB.

Moreover, a large number of clinical trials have assessed PARP inhibitors in combination with ICI therapy, most of which are still ongoing, including some in SCLC patients. Some available data have shown encouraging results of such combinations. In the MEDIOLA phase I/II basket trial, the combination of olaparib plus durvalumab (anti‐PDL1) represented a better disease control rate and improved survival in patients with germline BRCA (gBRCA)‐mutant metastatic breast cancer.^36^ In the same study, gBRCA‐mutant ovarian cancer patients had a high overall response rate (ORR).^36^ In SCLC, a prospective phase II single‐arm study reported that clinical benefit was observed in 21.1% (*n* = 4/19) of patients with confirmed responses or prolonged stable disease (8 months+).^37^ These clinical studies have shown that the therapeutic strategies including PARP inhibitors in combination with ICIs are very promising in many cancer types with HRD. Therefore, HRR gene mutation status has the potential to be a biomarker for response to this type of therapy in SCLC patients, in combination with other indicators (including TMB and PD‐L1).

This study has several limitations. We did not obtain prognostic data for the SCLC patients included in this study. For this reason, we could not reveal the relationship between HRD status and survival in the Chinese SCLC patients. Another limitation is that the sample size of our cohort was not large enough to assess germline HRR gene mutation patterns in Chinese SCLC patients and their influence on TMB and PD‐L1. These factors need to be assessed in further studies.

Our results have provided a comprehensive profile of HRR gene mutations in Chinese SCLC, illuminated the relationships of HRR gene mutation patterns with TMB and PD‐L1 and summarized the proportion of SCLC patients who might benefit from PARP inhibitors, ICIs or their combination. Nevertheless, more clinical trials need to be carried out to verify the predictive effect of HRR gene mutations on these treatment strategies.

## AUTHOR CONTRIBUTION

Shuo Wu, Yao Zhang, and Xin‐yu Ti conceived and designed the study. Yan Zhang and Xi Xu enrolled patients and prepared Fig's. Ying Du, Liz‐han Chen, Hai‐feng Ouyang managed, analyzed, and interpreted the data. Shuo Wu and Yao Zhang wrote the paper. Ying Du and Xin‐yu Ti critically revised the paper. All authors read and approved the final manuscript.

## ETHICS APPROVAL STATEMENT

The study was conducted with the approval of the Medical Ethics Committees of the First Affiliated Hospital of the Air Force Medical University (KY20202077‐C‐1).

## PATIENT CONSENT STATEMENT

Each patient signed a written informed consent form.

## Supporting information


Figure S1

Figure S2

Table S1

## Data Availability

The data and materials of the study are available from the corresponding author upon reasonable request.
