# Supplementary figures and images for: Mutational landscape of homologous recombination‐related genes in small‐cell lung cancer
Source: Cancer Med. 2022 Aug 26;12(4):4486–95. doi: 10.1002/cam4.5148 (PMC9972032; doi:10.1002/cam4.5148)

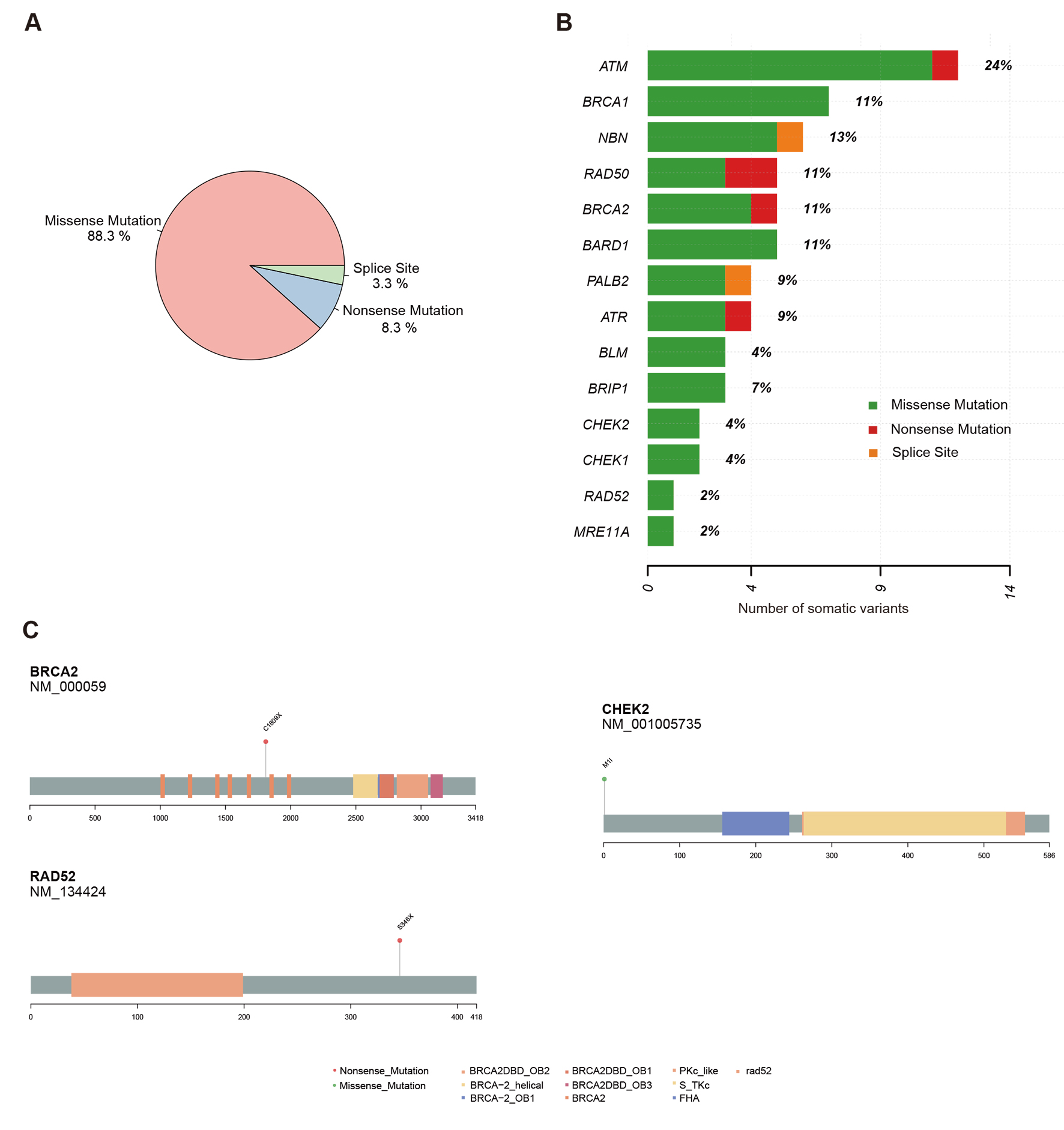

Supplement: Supplementary file 1 — Figure S1 [file CAM4-12-4486-s003.jpg]

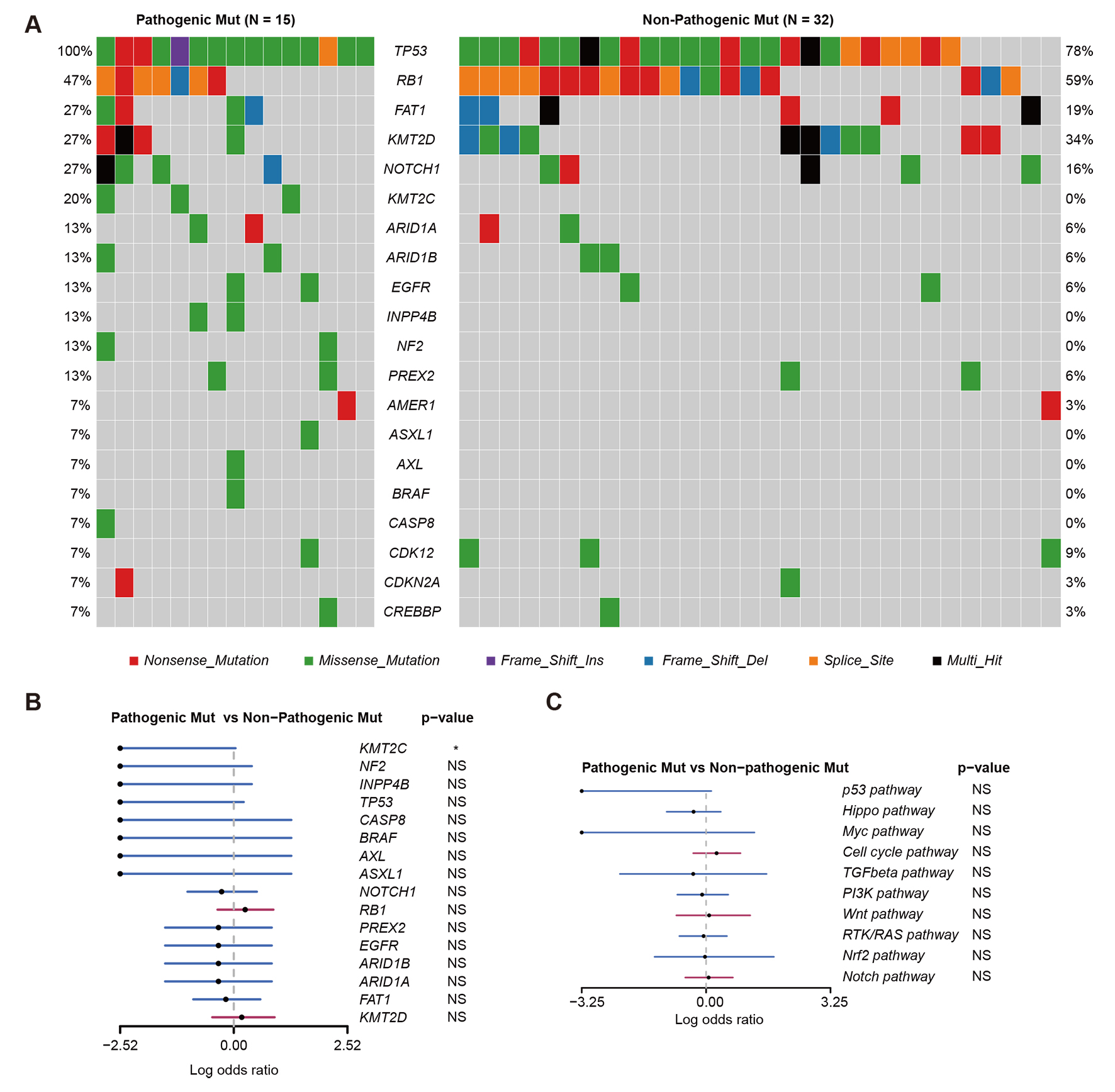

Supplement: Supplementary file 2 — Figure S2 [file CAM4-12-4486-s001.jpg]
