# Supplementary material for: Mutational landscape of homologous recombination‐related genes in small‐cell lung cancer
Source: Cancer Med. 2022 Aug 26;12(4):4486–95. doi: 10.1002/cam4.5148 (PMC9972032; doi:10.1002/cam4.5148)
Supplement: Supplementary file 3 — Table S1 [file CAM4-12-4486-s002.docx]

**TABLE S1** Pathogenic germline HRR gene variants in this cohort.

| Gene | Chromosome | Start  Position | End  Position | Variant  Classification | Transcript | Exon | Nucleotide  change | Amino acid  change |
| --- | --- | --- | --- | --- | --- | --- | --- | --- |
| RAD52 | chr12 | 1023218 | 1023218 | Nonsense | NM_134424 | exon11 | c.C1037A | p.S346X |
| CHEK2 | chr22 | 29130707 | 29130707 | Missense | NM_007194 | exon2 | c.G3A | p.M1I |
| BLM | chr15 | 91298040 | 91298040 | Splicing | NM_000057 | exon5 | c.960-1G>A | / |
| BRCA2 | chr13 | 32913919 | 32913919 | Nonsense | NM_000059 | exon11 | c.C5427A | p.C1809X |
